# Temporal Trends in Diagnostic Hemodynamics and Survival of Patients with Pulmonary Hypertension: A Single-Center Study

**DOI:** 10.3390/life13112225

**Published:** 2023-11-19

**Authors:** Georgios E. Papadopoulos, Alexandra Arvanitaki, Eleftherios Markidis, Sophia Anastasia Mouratoglou, Ioannis T. Farmakis, Panagiotis Gourgiotis, Thomas Chrysochoidis Trantas, Christos Feloukidis, Antonios Kouparanis, Matthaios Didagelos, Vasilis Grosomanidis, Antonios Ziakas, George Giannakoulas

**Affiliations:** Pulmonary Hypertension and Congenital Heart Disease Unit, AHEPA University Hospital, Aristotle University of Thessaloniki, 546 36 Thessaloniki, Greece; georgios.e.papadopoulos@gmail.com (G.E.P.); alexandra.arvanit@gmail.com (A.A.); markeleftherios@gmail.com (E.M.); s_mouratoglou@yahoo.gr (S.A.M.); gourgiopan@gmail.com (P.G.); thomas.chrysoch@gmail.com (T.C.T.); cfelouk@gmail.com (C.F.); akouparanis@gmail.com (A.K.); manthosdid@yahoo.gr (M.D.); grosoman@otenet.gr (V.G.); tonyziakas@hotmail.com (A.Z.)

**Keywords:** pulmonary hypertension, pulmonary arterial hypertension, hemodynamics, right heart catheterization, diagnosis, risk stratification, prognosis, survival, temporal changes

## Abstract

Advances in the diagnosis and treatment of pulmonary hypertension (PH) have gradually improved the disease course. This retrospective cohort study aims to explore the diagnostic hemodynamic profile and survival of PH patients and their temporal changes, as well as investigate potential prognostic factors. Overall, 257 adult patients were diagnosed with PH following right heart catheterization (RHC) from January 2008 to June 2023 according to the hemodynamic cut-off values proposed by the corresponding ESC/ERS guidelines at the time RHC was performed. Of these patients, 46.3% were Group 1, 17.8% Group 2, 14.0% Group 3, 18.0% Group 4, and 3.0% Group 5 PH. Temporal improvement in both diagnostic hemodynamic profile and survival of patients with PH and pulmonary arterial hypertension (PAH) was identified after 2013. Survival analysis demonstrated 5-year survival rates of 65% in Group 1 PH (90.3% in idiopathic PAH) and 77% in Group 4 PH. PAH patients being at low risk at diagnosis presented a similar 1-year all-cause mortality rate (12.4%) with high-risk ones (12.8%), primarily due to non-PH-related causes of death (62%), while high-risk patients died mostly due to PH (67%). The observed improvements in diagnostic hemodynamic profiles and overall survival highlight the importance of timely diagnosis and successful treatment strategies in PH.

## 1. Introduction

Pulmonary hypertension (PH) is characterized by elevated pulmonary artery pressure, associated with right heart dysfunction, impaired quality of life, and death, if left untreated. PH is hemodynamically defined as a mean pulmonary artery pressure >20 mmHg and is classified into five distinct groups, taking into account the underlying condition and clinical and hemodynamic features [1]. PH can be attributed to a wide spectrum of underlying conditions, such as left heart disease, lung disease, and pulmonary artery obstruction, but it can also be associated with drugs and toxins, connective tissue disease (CTD-PAH), HIV infection, portal hypertension (PoPH), congenital heart disease (CHD-PAH), and schistosomiasis, or it can be idiopathic (IPAH) or heritable (HPAH).

In 1987, the National Institutes of Health (NIH) registry for Pulmonary Artery Pressure [2] was pioneered, as the first landmark registry in PH, containing only patients with IPAH, with a diagnostic mean pulmonary artery pressure (mPAP) of 60 ± 18 mmHg. The first registry which included PH patients of various etiologies was held in Spain between 1998 and 2008 [3], reporting a diagnostic mPAP of 52.9 ±15.8 mmHg. Thirty years after the NIH registry, the Swedish registry [4] revealed a diagnostic median mPAP of 45 mmHg (IQR 16) in Groups 1 and 4 PH patients, which suggests an earlier diagnosis. 

Similar improvement has been reported in the survival of the disease. A US registry in 1991 [5], including only IPAH patients, demonstrated a 34% 5-year survival rate, while Boucly et al. [6] reported a 62% 5-year survival rate in 2021 in the same population. 

Consequently, advances in diagnostic strategies over the years resulted in diagnosing PH earlier, with most patients presenting a milder hemodynamic phenotype [7]. In addition, a proactive treatment approach enabled patients to live longer, frequently dying with the disease and not because of it [8,9]. Drawing from these successful initiatives, we present the results of a single PH-expert center study that compiles right heart catheterization (RHC) data, in tandem with demographic, clinical, laboratory, and survival characteristics of PH patients. This study aims to present the hemodynamic profile of PH patients at diagnosis and the profile’s temporal change over the years, as well as overall survival and survival temporal trends. 

## 2. Materials and Methods

This is a retrospective cohort that includes data from medical records of all patients who underwent RHC in our PH expert center from January 2008 to June 2023. This study has been approved by the Institutional Review Board of our center, in compliance with the Declaration of Helsinki. 

### 2.1. Inclusion Criteria

Patients ≥ 18 years of age who underwent RHC in the Catheterization Laboratory at AHEPA University Hospital of Thessaloniki, Greece, from January 2008 to June 2023 and who were diagnosed with PH were included. If the RHC was performed prior to the updated 2022 ESC/ERS guidelines for the diagnosis and treatment of PH patients [1], a mPAP of ≥25 mmHg was required to establish PH diagnosis with PCWP ≤ 15 mmHg and PVR ≥ 3WU for the pre-capillary PH; whether subsequent to the publication of the new guidelines, a mPAP > 20 mmHg established PH diagnosis with PCWP ≤ 15 mmHg and PVR > 2WU for the pre-capillary PH. We classified PH groups according to patients’ medical history, clinical presentation, echocardiography, pulmonary function testing, chest computed tomography (CT)-scans, and ventilation–perfusion imaging. 

### 2.2. Right Heart Catheterization

RHC was routinely performed via right jugular venous access. In patients with a congenital heart disease or difficulty in gaining jugular venous access, right femoral access was used. Mean right atrial pressure (mRAP); systolic and diastolic right ventricular pressure (sRVP and dRVP, respectively); systolic, diastolic, and mean pulmonary artery pressure (sPAP, dPAP, and mPAP, respectively); pulmonary capillary wedge pressure (PCWP); cardiac output/cardiac index (CO/CI); stroke volume/stroke index (SV/SVi); and mixed venous oxygen saturation (SvO2%) were measured. Thermodilution was the method of choice for the majority of patients when calculating CO [10]. In patients with an intra- or extra-cardiac shunt or severe tricuspid regurgitation, the indirect Fick method was used. Pulmonary artery compliance (PAC) was defined as PAC = SV/(sPAP − dPAP). Other parameters derived from the basic hemodynamics were also calculated (Appendix A).

### 2.3. Demographic and Clinical Data

Age, weight, height, body mass index (BMI), body surface area (BSA), heart rate at RHC, systolic blood pressure, hemoglobin, N terminal pro brain natriuretic peptide (NT-proBNP), and New York Heart Association Functional Class (NYHA class) were recorded before each RHC. The 6-min walking distance (6MWD) of the patients was recorded within a one-week interval of PH hemodynamic diagnosis. Survival data were collected until June 2023, either through telephone contact or via the national electronic health record, including cause of death, when available.

### 2.4. Temporal Trends in Diagnostic Hemodynamics and Survival

For the observation of temporal trends in diagnostic hemodynamics and survival, the cohort was divided into 5-year intervals, namely, January 2008–June 2013, July 2013–January 2018, and February 2018–June 2023. mPAP, PVR, and CO were the three hemodynamic variables that were compared among PH and PAH patients over time. The 1-year and 3-year survival rates were used to examine the temporal variance of the PH and PAH patients’ survival over the 5-year intervals. 

### 2.5. Risk Stratification

The 3-strata risk stratification model of the 2022 European Society of Cardiology (ESC)/European Respiratory Society (ERS) guidelines [1] was used to categorize PAH patients into a low, intermediate, or high-risk group. This was determined using the following parameters: NYHA class, 6-MWD, NT-proBNP, mRAP, Ci, stroke volume index (SVi), and SvO2% at baseline RHC. At least three variables were available for each patient. The European guidelines [1] proposed specific cut-off values ranging from 1 to 3, where 1 represented low risk, 2 denoted intermediate risk, and 3 indicated high risk. To ascertain the risk group for each patient, the sum of these grades was divided by the count of available variables. The resulting value was then rounded to the nearest whole number [11,12,13].

### 2.6. Statistics

Continuous variables following a normal distribution were presented as mean and standard deviation (SD), while variables that were not distributed normally were presented as median and interquartile range (IQR). The normality of a distribution was assessed by comparing the mean and median values, graphical representation of the distribution of the variables, and by using the Kolmogorov–Smirnov test. Qualitative variables were summarized using absolute and relative frequencies (n/N (%)). Statistical comparisons of continuous variables that exhibited a normal distribution were performed using the Student’s *t*-test, while the Wilcoxon rank-sum test was employed for variables that did not follow a normal distribution. Categorical variables were compared with the χ2 test or the Fisher exact test if cell counts were small (≤5). The Kruskal–Wallis test was used for a comparison of continuous variables between more than two independent samples. Mean values of mPAP, PVR, and CO in the total PH cohort, as well as in the PAH cohort, are depicted in a time plot to detect temporal changes of diagnostic hemodynamics. The Kruskal–Wallis test was used for a comparison among different time periods. The Kaplan–Meier method was used to estimate survival curves for all-cause mortality. The log-rank test was used to compare survival among PAH subgroups and between PAH and CTEPH. Survival trends were visualized in figures without formal statistical testing. All statistical analyses were performed using RStudio version 2023.03.0+386.

### 2.7. Missing Values

The k-nearest neighbors (k-NN) method was used to impute missing values when necessary. It is a non-parametric classification and regression algorithm used for pattern recognition and predictive modeling.

## 3. Results

### 3.1. Baseline Characteristics

Among the 294 patients who underwent RHC, 257 (87.4%) were diagnosed with PH. Overall, 119 patients (46.3%) were classified into Group 1, 46 (17.8%) into Group 2, 36 (14.0%) into Group 3, 47 (18.0%) into Group 4, and 9 (3.0%) into Group 5. A comprehensive overview of the baseline characteristics of patients within the various PH groups is presented in Table 1. At the point of diagnosis, Group 1 and Group 4 patients were younger compared to other groups. Cardiovascular comorbidities were present among all PH groups, with arterial hypertension and dyslipidemia affecting nearly half of PH patients (48% and 47%, respectively) and obesity being observed in one third of patients. Atrial fibrillation was highly prevalent in Group 2 (64%), while diabetes mellitus was equally prevalent in more than one third of patients in Groups 2 and 3, with a significant difference compared to the rest of the groups. No difference was detected in NYHA class, 6-MWD, or NT-proBNP at diagnosis among PH groups.

In terms of hemodynamics, patients with Group 2 PH exhibited higher mean right atrial pressure (mRAP) along with the lower pulmonary vascular resistance (PVR) compared to other PH groups. Group 1 patients had higher mixed venous oxygen saturation (SvO2), yet Group 4 encompassed patients characterized by lower CI, stroke volume index (SVi), and pulmonary artery compliance (PAC).

Baseline characteristics of PAH patients are presented in Table 2, highlighting distinct features within each subgroup. CHD-PAH patients were the youngest and presented less cardiovascular comorbidities, while patients with CTD-PAH and PoPH had the most favorable hemodynamic profile at diagnosis with a mPAP of 36 (IQR 11) mmHg and 32 (IQR 5) mmHg, respectively, and a PVR < 5 Wood Units in both groups. No difference among PAH subgroups was observed in NYHA class, 6-MWD, or NT-proBNP at diagnosis. At risk assessment during diagnosis, PAH patients were further classified into a low-risk group (21.2%), intermediate-risk group (56%), or high-risk group (22.8%) No difference in risk stratification was noted among PAH subgroups at diagnosis. 

### 3.2. Baseline Hemodynamic Data Variance over Time

**Figure 1:**
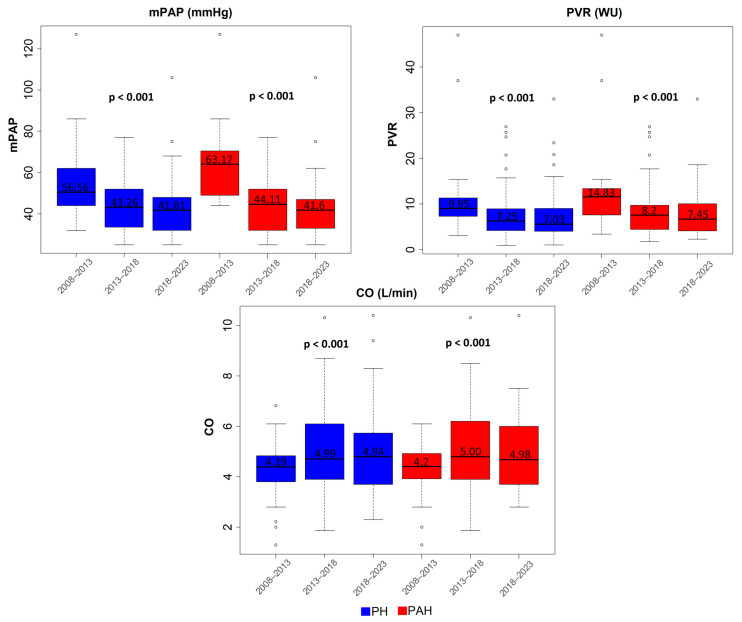
Hemodynamic data variance over time in PH and PAH patients. All hemodynamic variables significantly improved between the 2008–2013 and 2013–2018 periods and remained improved in 2018–2023. Abbreviations: mPAP: mean Pulmonary Artery Pressure, PVR: Pulmonary Vascular Resistance, CO: Cardiac Output, PH: Pulmonary Hypertension, PAH: Pulmonary Arterial Hypertension.

Figure 1 presents the temporal variance of the three hemodynamic variables at diagnosis among PH and PAH patients. mPAP and PVR at diagnosis presented a significant reduction in the 2013–2018 interval compared to 2008–2013, in both the PH and PAH cohorts, and remained decreased in 2018–2023. CO presented a significant increase from the 2008–2013 to 2013–2018 interval and remained increased in 2018–2023 in both the PH and PAH cohorts. 

### 3.3. Survival Temporal Trends

**Figure 2:**
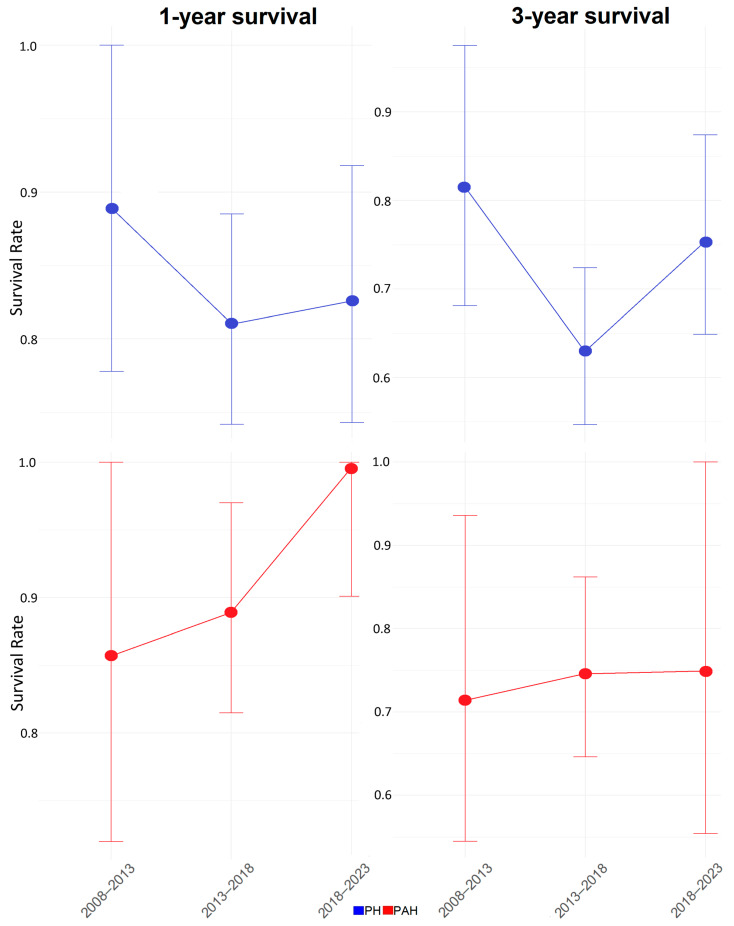
The 1-year and 3-year survival temporal trends in PH and PAH patients. Abbreviations: PH: Pulmonary Hypertension, PAH: Pulmonary Arterial Hypertension.

During a median follow-up duration of 5.3 years, 87 deaths were observed in the total PH cohort (0.06 deaths per patient-year). The 1-, 3-, and 5-year survival rates were 89.1%, 72.6%, and 62.8%, respectively. Figure 2 examines the temporal trends in 1-year and 3-year survival rates in the total PH cohort and separately in PAH. For PH patients, both 1-year and 3-year survival rates were better within the 2008–2013 time interval (89% and 81.5%, respectively) compared to the 2018–2023 interval. Recently diagnosed patients (in the 2018–2023 interval) had a better 1- and 3-year survival rate than those diagnosed between 2013 and 2018 (82.6% vs. 81% and 75.3% vs. 63%, 1-year and 3-year survival, respectively). On the contrary, PAH patients presented a gradual improvement in survival over the years, with 1-year survival rates of 85.7%, 88.9%, and 99.6% and 3-year survival rates of 71.4%, 74.6%, and 74.9% for the three 5-year intervals, respectively.

### 3.4. Survival Analysis of PAH Patients

**Figure 3:**
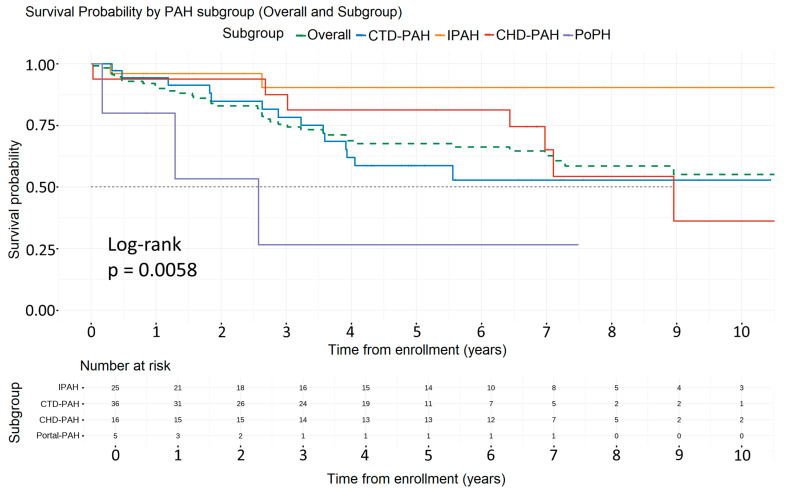
Survival analysis of the PAH patients for the entire cohort and PAH subgroups. Overall, 5-year mortality was 65% (green dashed line). CHD-PAH patients showed the better 5-year survival rate, 81.3%. Abbreviations: PAH: Pulmonary Arterial Hypertension, CTD-PAH: Connective Tissue Disease associated Pulmonary Arterial Hypertension, IPAH: Idiopathic Pulmonary Arterial Hypertension, CHD-PAH: Congenital Heart Disease associated Pulmonary Arterial Hypertension, PoPH: Portal Pulmonary Hypertension.

Figure 3 presents the Kaplan–Meier survival analysis among the different subgroups in PAH patients. During a median follow-up duration of 6.6 years, 41 deaths were observed (0.05 deaths per patient-year). The 1-, 3-, and 5-year survival rates for the PAH cohort were 91.6%, 77.7%, and 65%, respectively. The 5-year survival rates for PAH subgroups were as follows: IPAH (90.3%), CTD-PAH (58.7%), CHD-PAH (81.3%), and PoPH (27%) (*p*-value = 0.0058). Female patients exhibited superior survival compared to their male counterparts (5-year survival rates 75% vs. 55%, *p*-value = 0.0015). 

**Figure 4:**
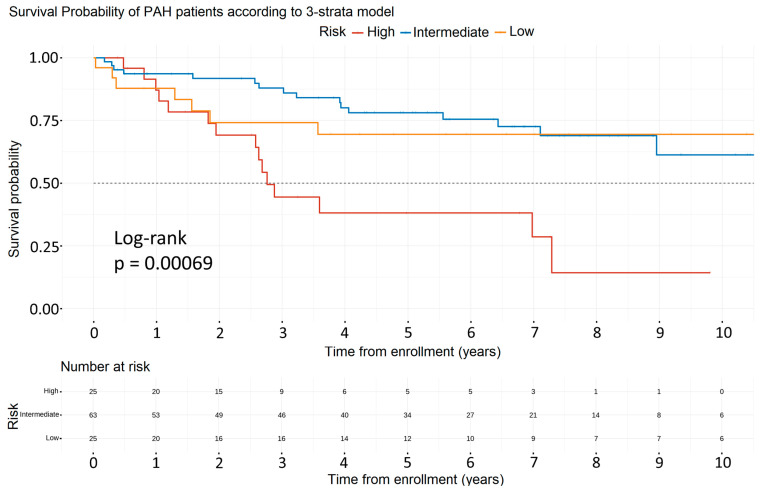
Survival analysis of the PAH patients among the different risk groups. Abbreviation: PAH: Pulmonary Arterial Hypertension.

The 1-year mortality risk within the low-risk, intermediate-risk, and high-risk groups was 12.4%, 6.7%, and 12.8%, respectively (*p*-value = 0.00069) (Figure 4). 

Table 3 examines the cause of death within each risk group. We observed that in the low- and intermediate-risk groups, the majority of deaths were non-PH-related (62% and 81%, respectively), unlike the high-risk group (33%, *p*-value = 0.027).

### 3.5. Survival Analysis of PAH and CTEPH Patients

**Figure 5:**
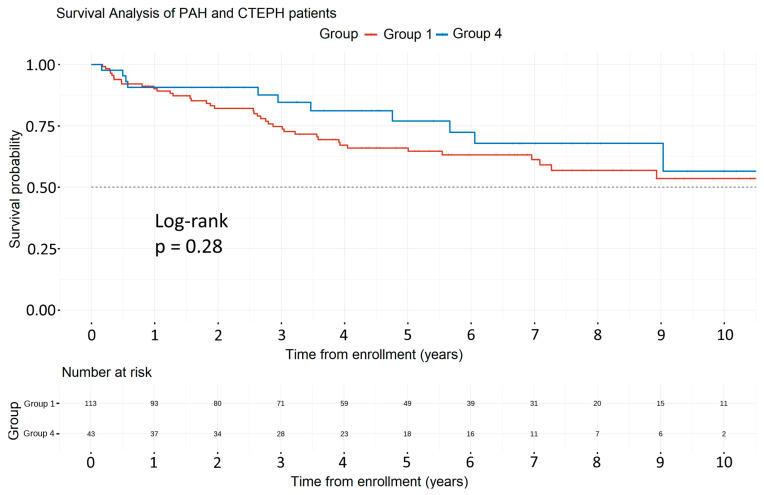
Survival analysis of PAH and CTEPH patients. Abbreviations: PAH: Pulmonary Arterial Hypertension, CTEPH: Chronic Thromboembolic Pulmonary Hypertension.

Figure 5 presents the Kaplan–Meier survival analysis comparing patients with PAH and CTEPH. During a median follow-up of 4.3 years, 11 CTEPH patients died (0.05 deaths per patient-year). The 5-year survival rate was 65% in PAH and 77% in CTEPH (*p*-value = 0.28).

## 4. Discussion

This single-center study summarizes the hemodynamic profile of patients with PH and PAH at diagnosis and demonstrates a substantial improvement in diagnostic hemodynamics over time. Furthermore, it presents the survival trends of various PH groups and PAH subgroups, depicting a temporal improvement in the survival of PAH patients, with those classified at low and intermediate 1-year mortality risk.

In detail, a substantial temporal improvement in the diagnostic hemodynamics of PH and PAH patients was demonstrated, particularly after 2013. Similarly, the Spanish and Swedish registries [3,4] revealed a significant improvement in the diagnostic hemodynamic profile of PH patients over the years. Table 4 summarizes the diagnostic hemodynamic data from previous registries compared to our cohort. The diagnostic hemodynamic profile of our patients aligns with previous registries and is clearly more favorable than the diagnostic profile reported in the oldest PH registry, REHAP [3]. REHAP included young PH patients with impaired hemodynamics, probably because CHD-PAH was the second-most prevalent subgroup.

Evolution of the diagnostic strategies, increased disease awareness, and the development of screening tools played a significant role in early PH diagnosis, even in asymptomatic mildly symptomatic patients, permitting early initiation of treatment and close follow-up. We observed that CTD-PAH and PoPH patients had the lowest diagnostic mPAP and PVR values among PAH patients. The close collaboration with rheumatology and hepatology clinics and the routine utilization of the DETECT algorithm as a screening tool for SSc patients may have contributed to this finding [17]. 

With regards to survival, encouraging data were reported in the temporal 1- and 3-year survival analysis of PAH patients, with survival in 2018–2023 outreaching the 1- and 3-year survival rates recorded in previous time intervals. On the other hand, PH patients presented a different pattern, with the most favorable survival rates observed in patients diagnosed between 2008 and 2013, while a notable improvement was detected in 2018–2023 compared to the 2013–2018 interval. This can be attributed to the comparatively fewer patients diagnosed with PH of different etiologies than PAH and the fewer deaths recorded during 2008–2013, which is not representative of the disease evolution. This study detected no significant difference in survival between PAH and CTEPH patients, as previously reported in the SWISS registry [16].

PAH patients demonstrated a similar 5-year survival rate of 65% compared to the 59% and 59.4% 5-year survival rates of the Swedish [4] and Giessen registries [15], respectively. Among PAH subgroups, patients with IPAH and CHD-PAH presented the best 5-year survival rates (90.3% and 81.3%, respectively), followed by the CTD-PAH cohort (58.7%). The corresponding rates reported in the Giessen registry [15] were lower, especially for the IPAH cohort (65.3%), which could be attributed to the chronological difference with the current study and the different treatment strategies utilized before 2011. Regarding CTD-PAH patients, we observed that despite the early diagnosis, they presented the most unfavorable outcome among PAH subgroups, following patients with portal hypertension, and this is consistent among many PH registries worldwide. 

The paradoxically higher 1-year mortality observed among PAH patients at low risk compared to those at intermediate risk led us to investigate their cause of death. We found that 62% of low-risk and 81% of intermediate-risk patients died due to non-PH-related causes. This finding can be explained by the fact that aggressive medical treatment may have prolonged survival in contemporary PAH patients and, therefore, non-PAH-related causes of death have emerged, especially in patients who are not at high risk at diagnosis. It is also known that most patients with PoPH die because of complications of their liver disease, while PAH directly or indirectly contributes to mortality in a minority of them [18]. Furthermore, the relatively low 1-year mortality rate (12.8%) in the high-risk group is quite encouraging compared to the >20% 1-year mortality risk suggested by the ESC/ERS guidelines [1] and could be attributed to the aggressive treatment strategy tailored to this population. 

Our study’s main limitation is the relatively small sample size. Secondly, the application of the average sum of points for each variable in calculating the risk score in PAH could be a potential limitation in our risk stratification methodology. While this approach is consistent with that employed in large European registries [11,12,13], it is important to acknowledge the ongoing debate regarding the relative importance of individual risk factors. The current ESC/ERS guidelines [1] do not explicitly prioritize specific parameters for risk classification. The single-center design of our study might also limit the generalizability of the findings to a broader population. However, it is important to note that our center serves as a referral center for PH patients from a broader region in northern Greece, mitigating the potential impact of selection bias. Lastly, the retrospective nature of this study might introduce bias related to data collection and patient selection. 

## 5. Conclusions

In conclusion, this single-center retrospective study offers a comprehensive perspective of the hemodynamic profile and survival of PH patients. The observed temporal improvements in diagnostic hemodynamic profiles and mortality rates highlight the evolving nature of PH care. Larger multi-center prospective cohorts are necessary to depict current diagnostic and management strategies and their impact on PH life expectancy.

## Figures and Tables

**Table 1:** Baseline characteristics of PH patients among different PH groups.

Characteristic	Overall, N = 257	Group 1, N = 119	Group 2, N = 46	Group 3, N = 36	Group 4, N = 47	Group 5, N = 9	*p*-Value ^1^
Age, years	63 (23)	59 (24)	72 (13)	71 (19)	60 (22)	61 (21)	**<0.001**
Sex Female	168/257 (65)	85/119 (71)	35/46 (76)	14/36 (39)	30/47 (64)	4/9 (44)	**0.002**
BMI, kg/m^2^	26.8 (7.6)	26.6 (7.3)	26.8 (8.6)	26.4 (6.0)	27.1 (9.6)	27.5 (7.3)	0.5
NYHA Class,							0.9
I	13/257 (5)	5/119 (3.9)	3/46 (7.1)	0/36 (0)	5/47 (11)	0/9 (0)	
II	125/257 (49)	51/119 (43)	33/46 (71)	15/36 (41)	22/47 (46)	4/9 (40)	
III	109/257 (42)	61/119 (51)	10/46 (21)	18/36 (50)	16/47 (34)	4/9 (40)	
IV	10/257 (4)	2/119 (2.0)	0/46 (0)	3/36 (9.1)	4/47 (8.6)	1/9 (20)	
6-MWD, m	389 (324, 483)	415 (331, 476)	384 (315, 426)	303 (170, 320)	396 (330, 489)	375 (263, 488)	0.14
NT-proBNP, pg/mL	544 (1293)	521 (1209)	866 (1115)	823 (1405)	381 (995)	775 (744)	0.8
NT-proBNP > 115 pg/mL *	147/167 (88)	75/85 (88)	17/19 (89)	21/23 (91)	33/38 (87)	1/2 (50)	0.5
Hypertension	77/160 (48)	33/82 (40)	15/22 (68)	12/21 (57)	15/31 (48)	2/4 (50)	0.2
Diabetes	38/161 (24)	20/85 (24)	8/22 (36)	8/21 (38)	2/29 (6.9)	0/4 (0)	**0.032**
Dyslipidemia	75/161 (47)	33/83 (40)	15/22 (68)	15/21 (71)	11/31 (35)	1/4 (25)	0.4
Atrial Fibrillation	29/157 (18)	6/83 (7.2)	14/22 (64)	5/19 (26)	4/29 (14)	0/4 (0)	**<0.001**
Obesity	82/255 (32)	39/118 (33)	19/46 (41)	7/36 (19)	15/46 (33)	2/9 (22)	0.3
Hemodynamics							
mRAP, mmHg	7.0 (6.0)	7.0 (6.0)	11.0 (7.0)	6.0 (5.3)	7.0 (6.5)	7.0 (1.0)	**<0.001**
mPAP, mmHg	42 (18)	42 (18)	42 (18)	38 (16)	43 (15)	43 (17)	>0.9
PVR, WU	6.3 (5.0)	6.7 (5.8)	4.3 (3.7)	7.5 (4.8)	6.7 (5.2)	4.8 (3.6)	**0.002**
PCWP, mmHg	11.0 (4.0)	10.0 (3.0)	18.0 (8.0)	10.0 (4.5)	12.0 (5.0)	10.0 (5.0)	**<0.001**
CI, mL/min/m^2^	2.5 (1)	2.6 (1)	2.8 (0.9)	2.3 (1.2)	2.2 (0.7)	2.9 (1.3)	**0.004**
SVi, mL/m^2^	34 (14)	34 (14)	37 (11)	31 (12)	29 (14)	40 (22)	**0.008**
SvO_2_, %	68 (11)	70 (10)	68 (11)	67 (11)	66 (10)	65 (6)	**0.033**
PAC, mL/mmHg	1.4 (1.3)	1.5 (1.4)	1.6 (1.1)	1.2 (1.2)	1.2 (0.7)	1.5 (1.9)	**0.029**

^1^ Kruskal–Wallis rank sum test; Fisher’s exact test. * The upper normal limit of the laboratory where NT-proBNP values were measured. Note: Continuous variables are presented as median value with interquartile range (IQR). Categorical variables are presented as n/N (%). Abbreviations: BSA: Body Surface Area, BMI: Body Mass Index, NYHA: New York Heart Association, 6-MWD: 6-Minute Walking Distance, NT-proBNP: N-terminal-pro brain natriuretic peptide, mRAP: mean Right Atrial Pressure, mPAP: mean Pulmonary Artery Pressure, PVR: Pulmonary Vascular Resistance, PCWP: Pulmonary Capillary Wedge Pressure, CO: Cardiac Output, Ci: Cardiac index, SV: Stroke Volume, SVi: Stroke Volume index, SvO_2_: Mixed Venous Oxygen Saturation, PAC, Pulmonary Artery Compliance.

**Table 2:** PAH patients’ baseline characteristics.

Characteristic	IPAH, N = 25	CTD-PAH, N = 37	CHD-PAH, N = 16	PoPH, N = 5	*p*-Value ^1^
Age, years	53 (27)	65 (13)	48 (25)	65 (3)	**0.008**
Sex Female	16 (64)	29 (78)	12 (75)	0 (0)	
BMI, kg/m^2^	30.8 (5.5)	26.4 (7.5)	24.3 (4.7)	30.9 (1.1)	**0.021**
NYHA Class,					0.6
I	1/25 (4.5)	1/37 (2.8)	0/16 (0)	0/5 (0)	
II	9/25 (36)	21/37 (58)	11/16 (69)	2/5 (40)	
III	13/25 (50)	12/37 (33)	4/16 (25)	3/5 (60)	
IV	2/25 (9.1)	2/37 (5.6)	1/16 (6.3)	0/5 (0)	
6MWD, m	367 (210, 450)	349 (211, 433)	430 (345, 475)	365 (240, 373)	0.5
NT-proBNP, pg/mL	521 (502)	749 (2179)	608 (2035)	197 (849)	0.3
NT-proBNP > 115 pg/mL *	18/21 (86)	32/34 (94)	13/15 (87)	3/5 (60)	0.14
Hypertension	11/25 (44)	21/37 (57)	3/15 (20)	2/5 (40)	0.11
Diabetes	11/25 (44)	5/37 (14)	1/15 (6.7)	2/5 (40)	**0.009**
Dyslipidemia	11/25 (44)	13/37 (35)	1/15 (6.7)	1/5 (20)	0.067
Atrial Fibrillation	1/25 (4.0)	4/37 (11)	2/15 (13)	0/5 (0)	0.7
Obesity	14/25 (56)	13/37 (35)	2/16 (13)	4/5 (80)	**0.008**
mRAP, mmHg	8.0 (6.0)	6.0 (4.0)	7.0 (3.3)	7.0 (5.0)	0.2
mPAP, mmHg	50 (12)	36 (11)	56 (15)	32 (5)	**<0.001**
PVR, WU	7.5 (5.0)	4.9 (4.5)	8.0 (9.3)	3.8 (1.4)	**0.005**
PCWP, mmHg	10 (4.00)	10 (4.00)	11 (2.00)	10 (3.00)	0.4
CI, L/m^2^	2.3 (0.8)	2.5 (1)	2.9 (1.2)	3.0 (1)	0.5
SVi, mL/m^2^	34 (15)	34 (13)	37 (13)	38 (14)	0.7
SvO_2_, %	66 (7)	71 (10)	77 (10)	71 (7)	**0.014**
PAC, mL/mmHg	1.4 (1.3)	1.7 (1.5)	1.3 (0.9)	2.7 (0.7)	0.11
Risk group					0.6
Low	4/25 (16)	9/37 (24)	4/16 (25)	1/5 (20)	
Intermediate	19/25 (76)	19/37 (51)	10/16 (63)	3/5 (60)	
High	2/25 (8.0)	9/37 (24)	2/16 (13)	1/5 (20)	

^1^ Kruskal–Wallis rank sum test; Fisher’s exact test. * The upper normal limit of the laboratory where NT-proBNP values were measured. Note: Continuous variables are presented as median value with interquartile range (IQR). Categorical variables are presented as n/N (%). IPAH subgroup contains HPAH and Drugs & Toxins PAH patients. Abbreviations: IPAH: Idiopathic Pulmonary Arterial Hypertension, CTD-PAH: Connective Tissue Disease associated Pulmonary Arterial Hypertension, CHD-PAH: Congenital Heart Disease associated Pulmonary Arterial Hypertension, PoPH: Portal Pulmonary Hypertension, BMI: Body Mass Index, NYHA: New York Heart Association, 6-MWD: 6-Min Walking Distance, NT-proBNP: N-terminal-pro brain natriuretic peptide, mRAP: mean Right Atrial Pressure, mPAP: mean Pulmonary Artery Pressure, PVR: Pulmonary Vascular Resistance, PCWP: Pulmonary Capillary Wedge Pressure, CO: Cardiac Output, Ci: Cardiac index, SV: Stroke Volume, SVi: Stroke Volume index, SvO_2_: Mixed Venous Oxygen Saturation, PAC, Pulmonary Artery Compliance.

**Table 3:** Cause of death in PAH patients among different 3-strata model risk groups.

	Low, N = 25	Intermediate, N = 66	High, N = 27	*p*-Value ^1^
All-cause mortality				**0.013**
Dead	8/25 (32)	17/64 (27)	15/25 (60)	
Cause of Death				**0.027**
PH-related	3/8 (38)	3/16 (19)	10/15 (67)	

^1^ Kruskal–Wallis rank sum test; Pearson’s chi-squared test; Fisher’s exact test; Note: Categorical variables are presented as n/N (%).

**Table 4:** Presentation of diagnostic hemodynamic data among different PH European registries.

Registry	AHEPA	REHAP [3]	SWEEDEN [4]	ASPIRE [14]	GIESSEN [15]	SWISS [16]
Period, years	2008–2023	1998–2008	2000–2014	2001–2010	1993–2011	1998–2012
Patients, Ν	257	1028	640	1344	1997	961
PH Classification						
Group 1 %	46	84	71	45	35	53
Group 2 %	18			12	15	4
Group 3 %	14			13	27	13
Group 4 %	18	16	29	18	23	26
Group 5 %	3			2		4
Demographics						
Age, years	63 (23)	47.5 (17.7)	67.9 (20)	59 (17)	60 (15)	60 (15)
Sex female, %	65.3	69.2	60.0	62.0	55.0	52.0
6MWD, m	389 (324, 483)	355 (120)	299 (218)		300 (120)	356 (138)
RHC						
mRAP, mmHg	7 (6)	9 (5)	7 (6)	11 (6)	7 (6)	9 (7)
mPAP, mmHg	42 (18)	52.9 (15.8)	45 (16)	45 (12)	42 (15)	46 (14)
PCWP, mmHg	11 (4)		8.6 (5)	13 (7)	10 (6)	12 (7)
PVR, WU	6.3 (5)	11.7 (5.9)	8.6 (5.7)	8.2 (5.4)	9.5 (7.5)	8.9 (5.6)
CI, mL/min/m^2^	2.5 (1.02)	2.6 (0.9)	2.3 (1)	2.7 (0.9)	2.3 (0.7)	2.5 (0.8)
SVO_2_, %	68 (11)		61.4 (13.2)	63 (9)	62 (9)	62 (10)

Note: Continuous variables are presented as median value with interquartile range (IQR) or mean (SD). Abbreviations: 6MWD: 6-Minute Walking Distance, mRAP: mean Right Atrial Pressure, PCWP, Pulmonary Capillary Wedge Pressure, PVR: Pulmonary Vascular Resistance, Ci: Cardiac index, SVO_2_: Mixed Venous Oxygen Saturation.

## Data Availability

The data presented in this study are available on request from the corresponding author. The data are not publicly available due to ethical restrictions.

## Appendix A

Parameters in the right heart catheterization (RHC) report that were calculated:

- Body Mass Index (BMI) = Weight × 10,000/(Height × Height)
- Body Surface Area (BSA) = 0.007184 × (Height(m)^0.725) × (Weight(kg)^0.425)
- Mean Pulmonary Artery Pressure (mPAP) = (2 × sPAP + dPAP)/3
- Pulmonary Vascular Resistance (PVR) = (mPAP − PAWP)/CO
- Stroke Volume (SV) = CO/HR
